# Chaigui granule exerts anti-depressant effects by regulating the synthesis of Estradiol and the downstream of CYP19A1-E2-ERKs signaling pathway in CUMS-induced depressed rats

**DOI:** 10.3389/fphar.2022.1005438

**Published:** 2022-10-24

**Authors:** Jun-sheng Tian, Peng-fei Qin, Teng Xu, Yao Gao, Yu-zhi Zhou, Xiao-xia Gao, Xue-mei Qin, Yan Ren

**Affiliations:** ^1^ Modern Research Center for Traditional Chinese Medicine of Shanxi University, Taiyuan, China; ^2^ Department of Psychiatry, First Hospital/First Clinical Medical College of Shanxi Medical University, Taiyuan, China; ^3^ Department of Psychiatry, Shanxi Bethune Hospital, Shanxi Academy of Medical Sciences, Tongji Shanxi Hospital, Third Hospital of Shanxi Medical University, Taiyuan, China; ^4^ Tongji Hospital, Tongji Medical College, Huazhong University of Science and Technology, Wuhan, China

**Keywords:** chaigui granule, depression, CYP19A1-E2-ERKs signaling pathway, chronic unpredictable mild stress, xiaoyao san

## Abstract

**Background**: There is a significant gender difference in the prevalence of depression. Recent studies have shown that estrogen plays a crucial role in depression. Therefore, studying the specific mechanism of estrogen’s role in depression can provide new ideas to address the treatment of depression. Chaigui granule has been shown to have exact antidepressant efficacy, and the contents of saikosaponin (a, b_1_, b_2_, d) and paeoniflorin in Chaigui granule are about 0.737% and 0.641%, respectively. Some studies have found that they can improve depression-induced decrease in testosterone (T) levels (∼36.99% decrease compared to control). However, whether Chaigui granule can exert antidepressant efficacy by regulating estrogen is still unclear. This study aimed to elucidate the regulation of estrogen levels by Chaigui granule and the underlying mechanism of its anti-depressant effect.

**Methods**: Eighty-four male Sprague-Dawley (SD) rats were modeled using a chronic unpredictable mild stress (CUMS) procedure. The administration method was traditional oral gavage administration, and behavioral indicators were used to evaluate the anti-depressant effect of Chaigui granule. Enzyme-linked immunosorbent assay (ELISA) was adopted to assess the modulating impact of Chaigui granule on sex hormones. Then, reverse transcription-quantitative PCR (RT-qPCR), and Western blot (WB) techniques were employed to detect extracellular regulated protein kinases (ERK) signaling-related molecules downstream of estradiol in the hippocampus tissue.

**Results**: The administration of Chaigui granule significantly alleviated the desperate behavior of CUMS-induced depressed rats. According to the results, we found that Chaigui granule could upregulate the level of estradiol (E2) in the serum (∼46.56% increase compared to model) and hippocampus (∼26.03% increase compared to model) of CUMS rats and increase the levels of CYP19A1 gene and protein, which was the key enzyme regulating the synthesis of T into E2 in the hippocampus. Chaigui granule was also found to have a significant back-regulatory effect on the gene and protein levels of ERβ, ERK1, and ERK2.

**Conclusion:** Chaigui granule can increase the synthesis of E2 in the hippocampus of CUMS-induced depressed rats and further exert antidepressant effects by activating the CYP19A1-E2-ERKs signaling pathway.

## 1 Introduction

The high prevalence and the heavy social burden of depression have attracted increasing attention (Huang et al., 2019; Papathanasiou et al., 2020). Some studies have shown that women are more likely to suffer from depression than men, approximately 1.5–3 times more often than men (Nelemans et al., 2021). Among depressed patients, depressive episodes last longer and recur more frequently in women (Walf and Frye, 2006). While the reasons for vulnerability to such disorders in women remain to be fully understood, the strongest candidate is the disruption of gonadal steroid levels (Albert and Newhouse, 2019). Studies have found that estrogen therapy can produce anti-depressant effects (Saad et al., 2019). The use of estrogen can be accompanied by severe adverse reactions, including coronary heart disease, stroke, and breast cancer (Rozenberg et al., 2013). In recent years, traditional Chinese medicine has unique advantages in the treatment of depression, with multiple components, multi-target, multi-mechanism, fewer side effects, strong synergy, and overall regulation. Therefore, traditional Chinese medicine is expected to become an effective and safe treatment option for patients with depression.

Chaigui granule is derived from the traditional Chinese medicine Xiaoyao San, which is composed of Mentha *canadensis* L. [Lamiaceae; Mentha *canadensis* herba], Bupleurum chinense DC. [Apiaceae; Bupleurum chinense radix], *Glycyrrhiza* uralensis Fisch. ex DC. [Fabaceae; *Glycyrrhiza* uralensis radix], Angelica sinensis (Oliv.) Diels [Apiaceae; Angelica sinensis radix], Atractylodes macrocephala Koidz. [Asteraceae; Atractylodis macrocephalae rhizoma], and Paeonia lactiflora Pall. [Paeoniaceae; Paeonia lactiflora radix] in the dose ratio of 2:6:3:6:6:6. Modern pharmacological studies have proved that Xiaoyao San has a significant anti-depressant effect (Gao et al., 2021). Based on the apparent anti-depressant efficacy of Xiaoyao San, researches on the Chaigui granule, including the screening of the prescription, the assumption of effectiveness and safety, and the chemical composition analysis, were performed systematically (Wu et al., 2019). At present, as the preparation process of Chaigui granule has been optimized and the anti-depressant effect has repeatedly been proved in our previous work (Chen et al., 2016; Wu et al., 2019), it has met the approval as a potential novel anti-depressant drug by the National Medical Products Administration (No. 2018L03149). The multicenter, randomized, placebo-controlled Phase IIa clinical trial is performed in the National drug clinical trial centers, including the Sixth Hospital of Peking University, Oriental Hospital of Beijing University of Traditional Chinese Medicine, and Wuhan mental health center.

CYP19A1 is a cytochrome P450 family 19 subfamily 1 that is mainly found in gonadal and extragonadal tissues (others such as bone, adipose tissue, brain, *etc.*) (Yague et al., 2006). In recent years, numerous related studies have shown that CYP19A1 gene polymorphism is associated with depression (Ancelin et al., 2020). Experimental animal studies have shown that the elimination of CYP19A1 in female mice significantly decreases estrogen levels and depression-like behavior (Dalla et al., 2004). This may be related to the ability of CYP19A1 to convert T to E2 (Punjani et al., 2021). In addition, the hippocampus is the critical lesion in depression. It was reported that the granular cells of the dentate gyrus of the hippocampus carry an entire steroid production system that synthesizes steroid hormones from cholesterol (Rebourcet et al., 2020). Recent experiments provide evidence that hippocampal synthesis does occur, which suggests that hippocampal estrogen synthesis regulates estrogen-sensitive hippocampal responses (Scharfman and Maclusky, 2006). In our previous study on the anti-depressant effects of Chaigui granule, which has been confirmed that they restored the serum T levels of depressed rats (Chen et al., 2015). So, whether the antidepressant effect exerted by Chaigui granule is related to CYP19A1 promoting estradiol synthesis. However, there is no study on the effect of Chaigui granule on estradiol.

Estradiol, an anabolic steroid hormone, is the most biologically active estrogen in the human body (Kuiper, 1997; Russell et al., 2019). Many studies have found that E2 is closely associated with depression. Patients with depression showed a significant decrease in estradiol levels compared with normal subjects. Serum estradiol levels were significantly higher in treated depressed patients than before treatment (Baischer et al., 1995; Young et al., 2000; Furuta et al., 2013). The effect of E2 on depression is complex, and its mechanism of action is mainly related to estrogen receptors (ER). It has been reported (Scharfman and Maclusky, 2006) that E2 in the brain can activate the ERK-cAMP-response element binding protein (CREB) signaling pathway through binding to ER, thus exerting an anti-depressant role. At the same time, many clinical trials have shown that the ERKs signaling pathway is involved in the occurrence of depression, which indicates that ERKs signaling pathways are closely related to mental illness (Dwivedi et al., 2006; Duman et al., 2007; Yuan et al., 2010). In order to further clarify the specific mechanism of Chaigui granule exerting antidepressant efficacy, we investigated the effect of Chaigui granule on the levels of related sex hormones in the serum and hippocampus of CUMS rats, as well as the downstream pathways that may exert antidepressant efficacy. This provides a new idea and basis for traditional Chinese medicine to exert antidepressant efficacy by regulating sex hormones.

## 2 Materials and methods

### 2.1 Materials

Paeonia lactiflora Pall. (No.1806436111), Mentha *canadensis* L. (No.1802017131), Atractylodes macrocephala Koidz. (No.1809657151), Angelica sinensis (Oliv.) Diels (No.1803583111), Bupleurum chinense DC. (No.1805215131) and *Glycyrrhiza* uralensis Fisch. ex DC. (No.1810013171) as authenticated by Dr. Xuemei Qin from Modern Research Center for Traditional Chinese Medicine of Shanxi University. Chaigui granule were processed and prepared by the preparation center of Shanxi Academy of traditional Chinese medicine (No.20181009); HPLC analysis of Chaigui granule are shown in Supplementary Table S1; Venlafaxine Hydrochloride Capsule (No.181203) and Shuganjieyu Capsule (No.180101) were obtained from Chengdu Kanghong Pharmaceutical Group Co., Ltd.

### 2.2 Animals and ethic statement

84 male Sprague-Dawley (SD) rats, Specific Pathogen Free (SPF) grade, weight (180–200 g), were provided by Vital River Laboratory Animal Technology Co., Ltd. (Beijing) (SCXK-2016–0006). All animals were reviewed and approved by the Committee of Scientific Research at Shanxi University (CSRSX) in April 2018 (NO. SXULL2018015). Animal welfare and all experimental programs were carried out under the regulations of the State Committee of Science and Technology of the People’s Republic of China on the Administration of Experimental Animals. All animals were housed 5 per cage under controlled breeding room conditions (lights on at 8:00 a.m., 12 h light/dark cycle, temperature: 24 ± 2°C, humidity: 50% ± 20%.) with free access to food and water.

### 2.3 Procedure of CUMS-induced rats

**FIGURE 1:**
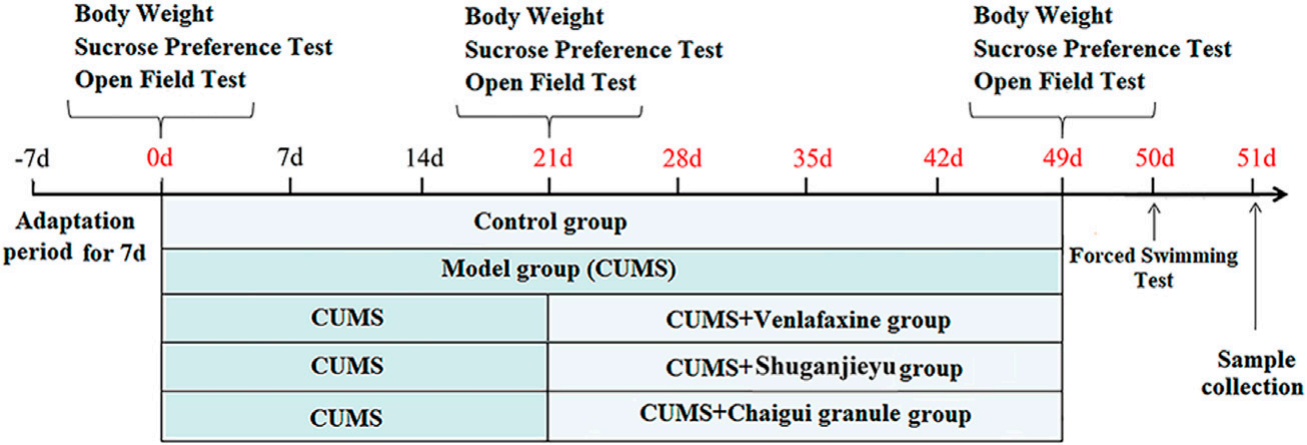
Schematic diagram of CUMS, drug treatment, and behavioral testing program schedule. Behavioral tests include sucrose preference test, open field test, and forced swim test.

Rats were randomly selected as control group (Con), model group (Mod), venlafaxine-treated group (35 mg/kg) (Ven), Shuganjieyu group-treated group (0.15 g/kg) (Shu), and Chaigui granule with high dose group (6.29 g drug/kg) (Hig), medium dose group (3.15 g drug/kg) (Med), low dose group (1.59 g drug/kg) (Low) respectively. Volume: 1 ml/100 g (rat body weight). The control and model groups were given normal saline 1 ml/100 g/d. CUMS model is improved by referring to the methods of Willner et al. (Willner et al., 1987). A stimulus factor was randomly selected every day, and the same stimulus factor could not appear continuously to ensure the randomness and unpredictability of the stimulus factor. The detailed schedule of the CUMS model is shown in Figure 1. Stressors used in the Chronic Unpredictable Stress paradigm are shown in Table 1.

**TABLE 1:** Stressors used in the Chronic Unpredictable Stress paradigm.

Stressor	Description
thermal stimulus	10 min at 45°C
ice-water bath	4°C ice water bath for 5 min
ultrasonic stimulation	Power: 60 W, 3 h
tail pinch	Clamp the tail root of the rat about 1 cm for 2 min
circadian disorder	Rats were treated with 24 h of light exposure
foot shock	Electric shock at 36 V voltage every 10 s, lasting for 2 s each time, 10 times in total
water deprivation	Water was deprived for 24 h
restrain	The rats were fixed for 3 h, so that their limbs could not move freely
fasting	Feed deprivation for 24 h

### 2.4 Behavior test

#### 2.4.1 Body weight

During the CUMS induction period, the body weights of rats were recorded regularly throughout the experiment, and all rats in each group were weighed at 0, 3, and 7 weeks. This was used to evaluate CUMS modeling and the effect on rat body weight after administration.

#### 2.4.2 Sucrose preference test (SPF)

Rats were given a bottle of purified water and a bottle of 1% sucrose water. Purified water was fixed on the right side, and sucrose water was fixed on the left side. Sugar water preference training was then performed for 24 h, and the positions of the two bottles were exchanged at 12 h. After the sugar water training, a sugar water preference test was performed for 3 h. Sugar water preference was calculated by measuring the weight of the bottles before and after the experiment (Qi et al., 2018). Sugar water preference rate = (sucrose water consumption/total water consumption) × 100%.

#### 2.4.3 Open field test (OFT)

A self-made open-field box (5 × 5) was used to place the rat pinto in the central grid and adapt for 1 min. Record the activity of the rat in the open field for 4 min, including recording the stay time in the central grid, the number of crossing grids, and the number of standing upright (Sturman et al., 2018). The whole experimental process is under normal lighting conditions, and noise interference is avoided. After the measurement of each rat, the open field box will be cleaned and sprayed with alcohol before the measurement of the next rat.

#### 2.4.4 Forced swimming test (FST)

On the first day after the experiment (the 50th day), each rat was placed in a 10 L glass beaker containing clean water at 25 ± 1°C, filled to a depth of 20 cm. The process took 6 mins, and the rat’s immobile time was recorded for 4 min. Immobility was defined as no movement of limb or body, including staying afloat, except movements caused by staying afloat (Eid et al., 2020).

### 2.5 *Hippocampus* and serum samples collection and preparation

#### 2.5.1 Serum sample

After the end of the experiment, the blood of rats was collected, placed in the blood collection vessel without heparin sodium, after standing for 30 min, centrifuge at 3500 r/min for 14 min at 4°C, and the serum was separated and divided into 2.5 ml EP tubes, and stored in the refrigerator at -80°C.

#### 2.5.2 *Hippocampus* sample

After the end of the experiment, the rat brain was quickly dissected on ice, and the hippocampus samples were separated and placed in a 1.5 ml cryopreserved tube. The samples were quickly frozen in liquid nitrogen and stored in a refrigerator at -80°C.

### 2.6 Biochemistry assay

#### 2.6.1 Enzyme-linked immunosorbent assay (ELISA)

Rat testosterone ELISA kit (No.F16854), Rats estradiol ELISA kit (No.F15321), and Rat aromatase P450 19A1 ELISA kit (No.F11248) were obtained from Sangon Biotech (Shanghai) Co., Ltd. and performed according to the manufacturer’s instructions.

#### 2.6.2 Reverse transcription-quantitative polymerase chain reaction

Total RNA was extracted from hippocampal tissues by referring to the specification of EZ-10RNA Miniprep kits (No.B618133, Sangon Biotech (Shanghai) Co., Ltd.). A multi-functional enzyme marker determined the OD260/280 value and OD260/230 value of RNA. The OD260/280 of RNA ranged from 1.8 to 2.0, and OD260/230 was more significant than 2.0, indicating that the purity of RNA was good. Then configure the reverse transcription system (20 μl reaction system) according to the instructions of the M-MuLV First Strand cDNA Synthesis Kit (No.b532435, Sangon Biotech (Shanghai) Co., Ltd.) to reverse transcribe mRNA into cDNA and perform the reaction on ice. Finally, prepare the PCR reaction solution on ice according to the instructions of the SGExcel FastSYBR Mixture (No. B532955, Sangon Biotech (Shanghai) Co., Ltd.). The PCR reaction procedure is pre-denaturation at 95°C for 3 min, then denaturation at 95°C for 5 s, annealing and extension at 60°C for 20 s, and a total of 40 cycles. Each sample of RT-qPCR was repeated in three replications. The primer sequence is shown in Table 2.

**TABLE 2:** Key target primer sequences of CYP19A1-E2-ERK1/2 signaling pathway.

Number	Target	Primer_F	Primer_R
**1**	GADPH	CAA​GTT​CAA​CGG​CAC​AGT​CAA	CGC​CAG​TAG​ACT​CCA​CGA​CA
**2**	AR	CAGTAGCCCAAGCGATGC	TCC​CTG​GTA​CTG​TCC​AAA​CG
**3**	CYP19A1	CAC​TTC​TAA​CAC​GCT​CTT​CCT​G	GCA​AAA​TCC​ATA​CAG​TCT​TCC​A
**4**	ERα	TGG​ACA​GGA​ATC​AAG​GTA​AAT​G	CAG​GAC​TCG​GTG​GAT​GTG​G
**5**	ERβ	AGC​ACC​TTG​AGT​CCA​GAG​CA	CAG​TCC​CAC​CAT​TAG​CAC​CT
**6**	ERK1	TCC​CAA​ATC​TGA​CTC​CAA​AGC	TCCTTCAGCCGCTCCTTG
**7**	ERK2	ATC​ACA​TCC​TGG​GTA​TTC​TTG​G	GAG​CTT​TGG​AGT​CAG​CGT​TT

#### 2.6.3 Western blot analysis

Western blot was used to analyze the levels of AR, CYP19A1, ERα, ERβ, ERK1, and ERK2 proteins in the hippocampus. Put 30 mg of hippocampus tissue into a 1.5 ml EP tube, add 150 μl of RIPA lysate mixture (PMSF: RIPA = 1:100), ground with tissue grinder for 2 min, then put it into ice water for 1 h. During this period, took it out and shook it several times to ensure that the tissue was fully lysed. After centrifugation at 13,000 rpm for 15 min at 4°C, take the supernatant to obtain the total protein solution. Then, that protein concentration was determined using a BCA protein detection kit (No.M1003, miniBio). Each sample of the protein concentration was quantitatively set at 2.5 mg/ml, and the prepared protein samples were boiled in a 99°C metal bath for 10 min and then stored at −80°C. The protein samples were separated by 8% SDS-PAGE (No.M1014, minibio) and transferred onto polyvinylidene difluoride membranes, and then blocked with TBST sealant (5% skimmed milk powder) at 37°C. Select the desired strips on the transferred PVDF membrane, and place them in the corresponding primary antibody diluent. After complete incubation, place the washed target strip in the diluent of the secondary antibody and react on a shaker at 37°C for 1 h. After total incubation, remove the target strip and wash it with TBST 3 times. Rabbit Anti-Estrogen Receptor alpha Polyclonal Antibody (No.bs-0122R, Bioss); Rabbit Anti-Androgen Receptor Polyclonal Antibody (No.bs-0118R, Bioss); Rabbit Anti-Estrogen Receptor beta Polyclonal Antibody (No.bs-0116R, Bioss); P44/42 MAPK (ERK1/2) Rabbit mAb (No.137F5, Cell Signaling); Phospho-P44/42 MAPK (ERK1/2) (Thr202/Tyr204) Rabbit mAb (No.D13.14.4E, Cell Signaling); Anit-Aromatase antibody (EPR4532-2) (No.ab124776, Abcam); GAPDH Rabbit mAb (No.D16H11, Cell Signaling); The mime color protein marker is 10–180kda (No.M1028, minibio);

### 2.7 Statistical analysis

The 2^−ΔΔCt^ method was used to calculate the quantitative real-time PCR results (Livak and Schmittgen, 2013). The experimental data were expressed as mean ± standard deviation (mean ± SD). One-way ANOVA was used for comparison between groups, and SPSS 22.0 was used for statistical analysis. The results of ANOVA were *p* < 0.05 or *p* < 0.01.

## 3 Result

### 3.1 The anti-depressant effects of chaigui granule on behavior

**FIGURE 2:**
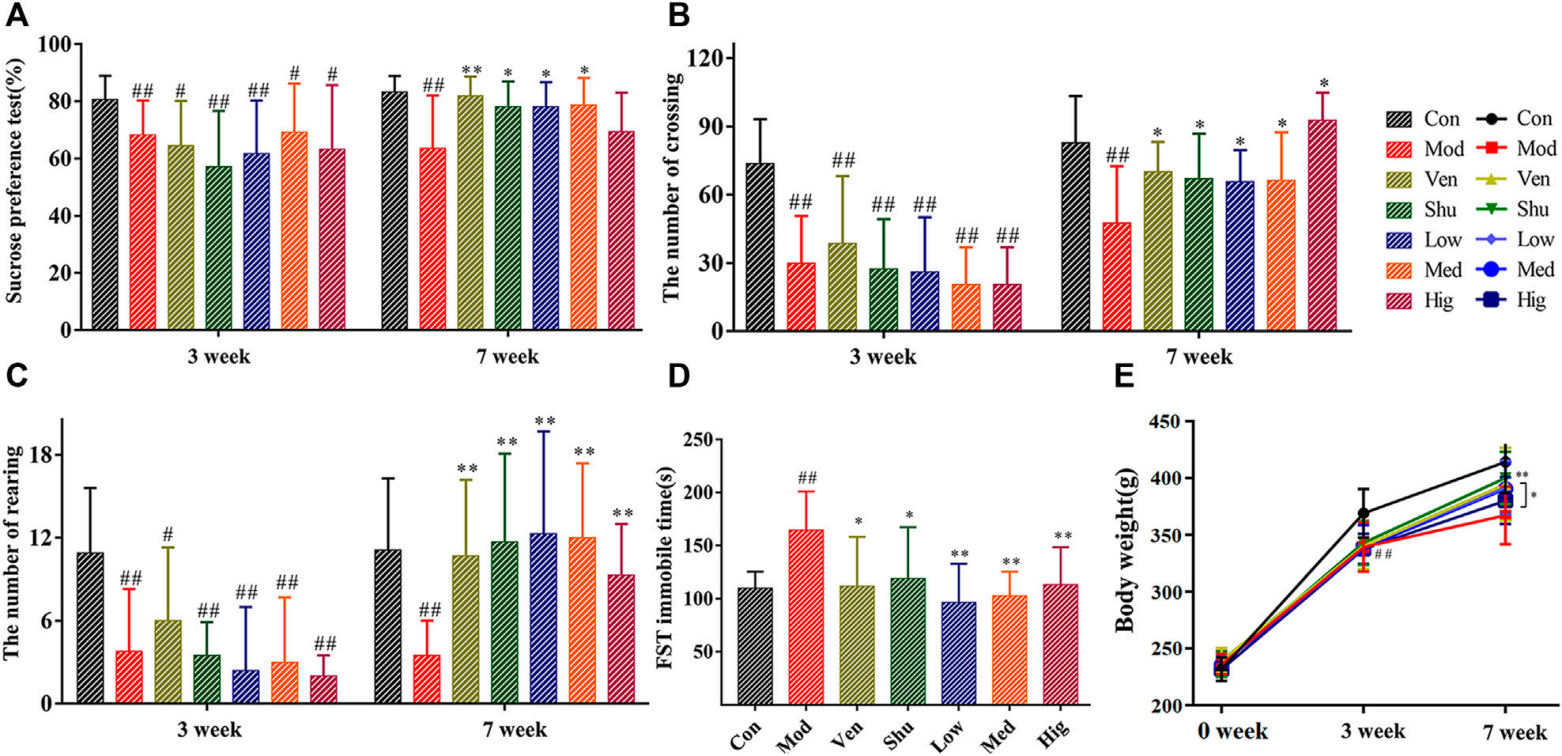
The effect of Chaigui granule on behavior. **(A)** Sucrose preference (%). **(B)** The number of crossings. **(C)** The number of rearing. **(D)** Immobile time of FST. **(E)** Body weight (g). All data were expressed as mean ± SD. ^#^ *p* < 0.05, ^##^ *p* < 0.01 compared with Control group. ^✻^ *p* < 0.05, ^✻✻^ *p* < 0.01 compared with Model group.

The behavioral results indicated that the rat model of depression was successfully replicated after 3 weeks of modeling (Figure 2). Treatment was administered in the following 4 weeks of modeling, and analysis of behavioral data revealed that all treated groups could improve depression-like behavior in CUMS rats. Compared with the CUMS group, the sucrose preference rate (Figure 2A) was significantly increased after treatment with Chaigui granule, Ven (positive group), and Shu (positive group) at the 7 weeks, indicating significant improvement in the anhedonia and reward behavior dysregulation in CUMS model rats. The crossing number (Figure 2B) and the rearing number (Figure 2C) were also significantly increased after the treatment at the 7 weeks, indicating the improvement of mobility reduction, reduction of acting ability, and capability of exploring new things in the CUMS rats. However, the immobility time was significantly decreased in the FST (Figure 2D), indicating that desperate behavior in CUMS model rats was improved and regulated considerably. At the 7 weeks, the body weights of rats in the Chaigui granule, Ven, and Shu groups were significantly increased compared with the CUMS group (Figure 2E). These results indicated that the Chaigui granule remarkably improved the depressive-like behavior of CUMS-induced rats and had notably anti-depressant effects.

### 3.2 Regulation of Chaigui granule on sex hormone in serum and hippocampus of CUMS-induced depressed rats

**FIGURE 3:**
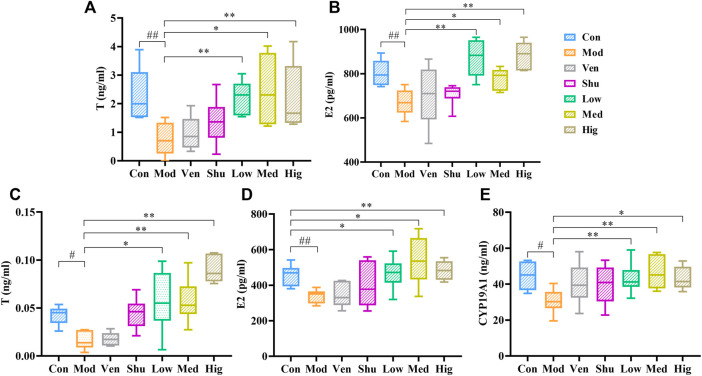
The effect of Chaigui granule on sex hormones. **(A)** The T (mg/ml) levels in serum. **(B)** The E2 (pg/ml) levels in serum. **(C)** The T (mg/ml) levels in hippocampus. **(D)** The E2 (pg/ml) levels in hippocampus. **(E)** The CYP19A1 (ng/ml) level in hippocampus. All data were expressed as mean ± SD. ^#^ *p* < 0.05 compared with Control group. ^✻^ *p* < 0.05, ^✻✻^ *p* < 0.01 compared with Model group.

Previous studies have found that Chaigui granule can reverse the level of T in the serum of depressed rats (Chen et al., 2015). However, Sex hormone levels in the brain are not only related to self-synthesis but also to peripheral sex hormone levels, which is based on the fact that sex hormones can cross the blood-brain barrier (Cui et al., 2013). In order to investigate the potential antidepressant mechanism of Chaigui granule and whether it also regulates E2, we measured the contents of T and E2 in the serum and hippocampus of experimental rats. Compared with the control group, T and E2 were lower in both serum and hippocampus in the CUMS group (Figures 3A–D), suggesting that CUMS modeling could lead to disturbance of sex hormone levels in rats. In contrast, the levels of T and E2 in serum and hippocampus were significantly increased after gavage with Chaigui granule (Figures 3A–D). These results showed that Chaigui granule had a significant inhibitory effect on the decrease of T and E2 contents in serum and hippocampus induced by CUMS rats.

Based on the above findings and previous literature research, we are very interested in how the Chaigui granule regulates E2 levels in the hippocampus of depressed rats. Next, we examined CYP19A1 content in the hippocampus of experimental rats. It was found that the content of CYP19A1 in the hippocampus of CUMS group was also lower than in the control group (Figure 3E); however, after treatment, Chaigui granule could significantly inhibit the decrease of CYP19A1 level in the hippocampus of rats induced by CUMS modeling. However, venlafaxine and Shuganjieyu capsule did not show regulatory effects on T, E2, and CYP19A1 in the serum or hippocampus of treated rats. Therefore, the regulatory impact of Chaigui granule on T, E2 and CYP19A1 content may be a potential mechanism of its antidepressant effect.

### 3.3 The mRNA and protein levels of the CYP19A1-E2-ERK1/2 pathways in the hippocampus

**FIGURE 4:**
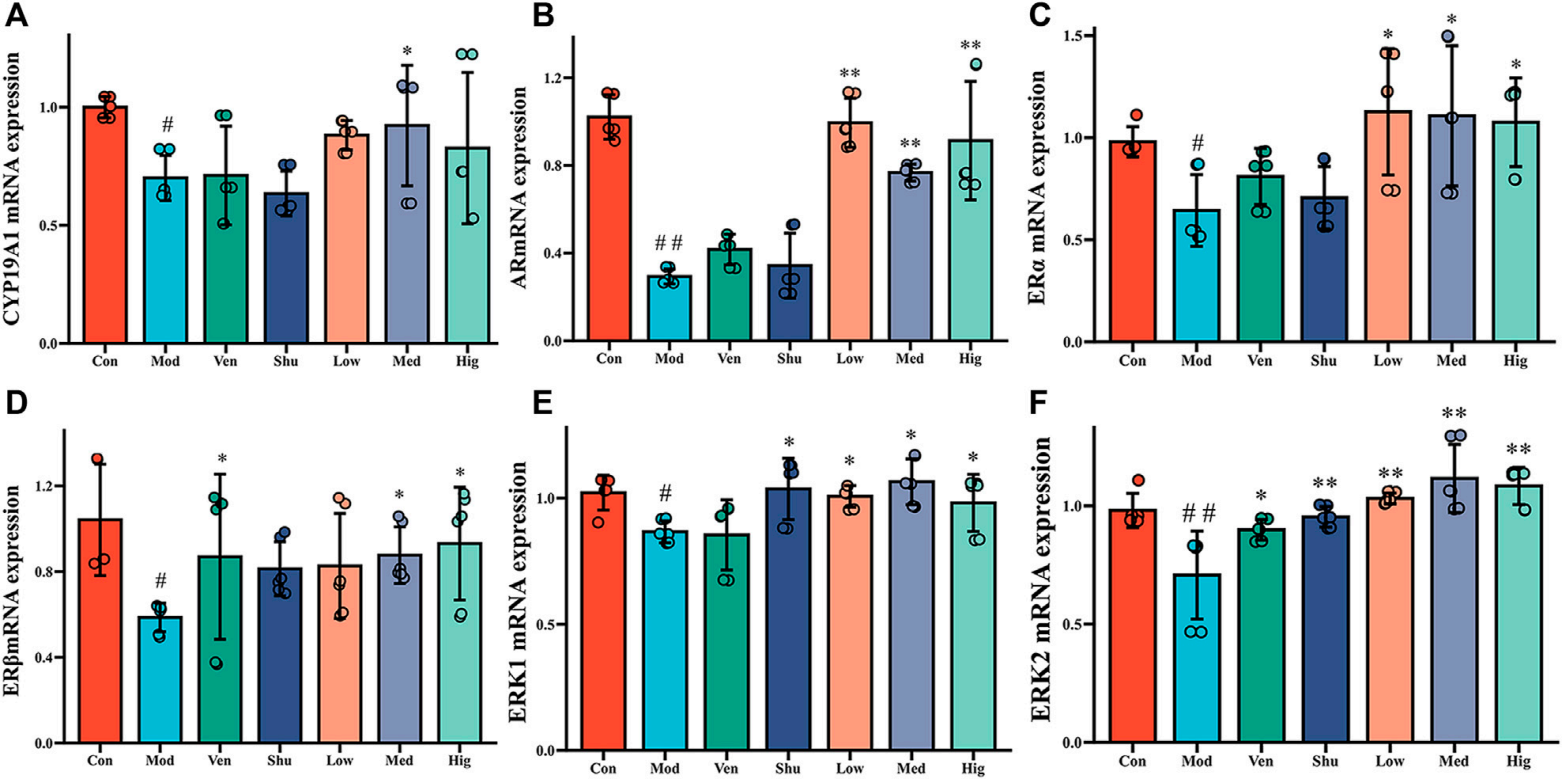
The effect of Chaigui granule on mRNA expression of CYP19A1-E2-ERK1/ERK2 pathway in the hippocampus. **(A)** The expression of CYP19A1 mRNA in the hippocampus. **(B)** The expression of AR mRNA in the hippocampus. **(C)** The expression of ERα mRNA in the hippocampus. **(D)** The expression of ERβ mRNA in the hippocampus. **(E)** The expression of ERK1 mRNA in the hippocampus. **(F)** The expression of ERK2 mRNA in the hippocampus. All data were expressed as mean ± SD. ^#^ *p* < 0.05, ^##^ *p* < 0.01 compared with Control group. ^✻^ *p* < 0.05, ^✻✻^ *p* < 0.01 compared with Model group.

*Hippocampus* is the critical lesion in depression. According to previous studies (Prange-Kiel et al., 2003; Rune and Frotscher, 2005), the estradiol level in the brain can play an anti-depressant role. To further investigate whether Chaigui granule exerts antidepressant efficacy by regulating estradiol and its downstream signaling pathways, we measured ERKs signaling pathways downstream of E2 using RT-qPCR and WB techniques. It was shown that the mRNA levels of CYP19A1, AR, ERα, ERβ, ERK1, and ERK2 mRNA in the hippocampus of CUMS group were significantly reduced compared with the control group. In contrast, the levels of CYP19A1 mRNA in the hippocampus were significantly increased after administration of Chaigui granule compared with the CUMS group (Figure 4A). Positive drugs Ven and Shu had no ameliorating effect But had a regressive impact on hippocampal ERβ mRNA with no pronounced difference. The levels of AR and ERα and ERβ mRNA in the hippocampus were significantly increased after administration of Chaigui granule and Ven compared with the CUMS group (Figures 4B–D). Positive drugs Ven and Shu had no improvement effect on the AR or ERα mRNA in the hippocampus but had a regressive effect on hippocampal ERβ mRNA. The levels of ERK1 and ERK2 mRNA in the hippocampus were significantly increased after the administration of Chaigui granule, Shu, and Ven compared with the CUMS group (Figures 4E,F).

**FIGURE 5:**
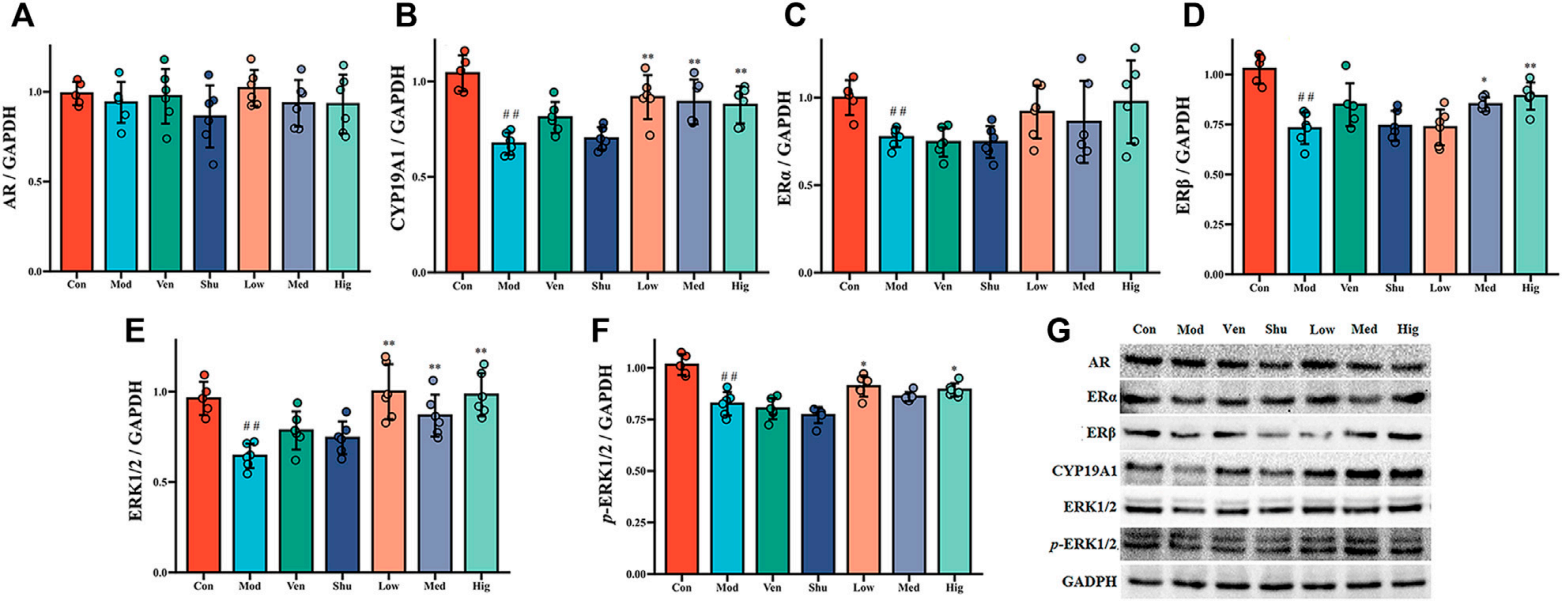
The effect of Chaigui granule on protein expression of CYP19A1-E2-ERK1/ERK2 pathway in the hippocampus. **(A)** The protein expression of AR in the hippocampus. **(B)** The protein expression of CYP19A1 in the hippocampus. **(C)** The protein expression of ERα in the hippocampus. **(D)** The protein expression of ERβ in the hippocampus. **(E)** The ratio of ERK1 and ERK2 protein expression in the hippocampus. **(F)** The ratio of p-ERK1 and p-ERK2 **(G)** protein expression in the hippocampus. All data were expressed as mean ± SD. ^#^ *p* < 0.05, ^##^ *p* < 0.01 compared with Control group. ^✻^ *p* < 0.05, ^✻✻^ *p* < 0.01 compared with Model group.

Compared with the control group, the protein levels of CYP19A1, ERα, ERβ, ERK1/2, and p-ERK1/2 in the hippocampus of CUMS group were significantly reduced. The protein levels of CYP19A1, ERα, and ERβ in the hippocampus were significantly increased after the treatment (Figures 5B–D). The protein levels of ERK1/2 and p-ERK1/2 in the hippocampus were also considerably increased after the Chaigui granule and Ven administration compared with the CUMS group (Figures 5E,F). These results suggest that Chaigui granule can not only regulate the content of E2 in the hippocampus of CUMS rats but also regulate its downstream ERKs signaling pathway from two levels: gene and protein, thus exerting antidepressant effects.

**FIGURE 6:**
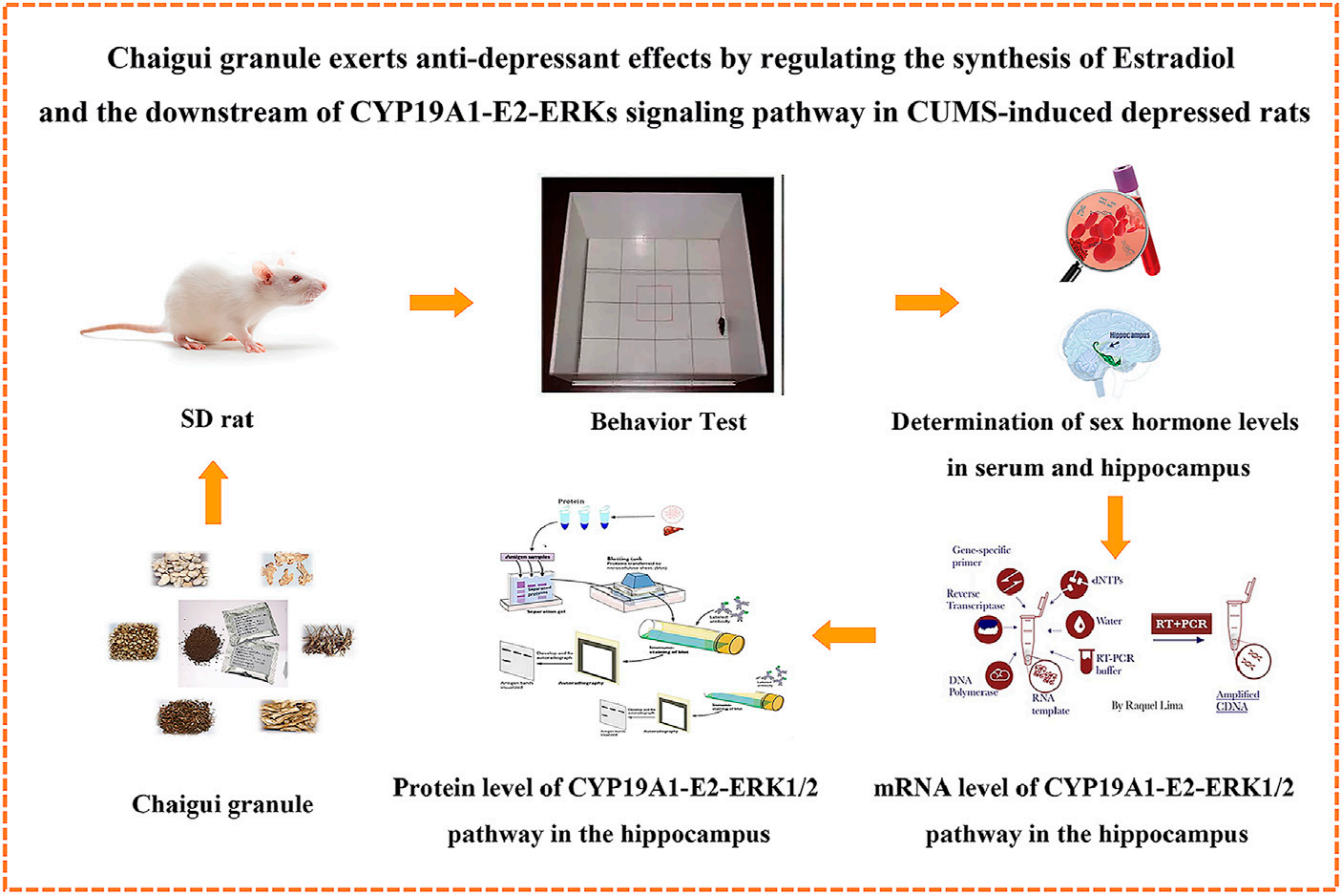
Graphic abstract.

## 4 Discussion

Depression is a psychiatric disorder with a high prevalence and high disability rate. Although the current clinical estrogen therapy is effective, it is also associated with many side effects. Chaigui granule is a new type of anti-depressant drug. In the previous experiment, the composition of the Chaigui granule was further studied and 95 compounds were explicitly identified. This provides a basis for the study of the chemical composition of the Chaigui granule (Xg et al., 2020). Combined with the previous research, the Chaigui granule also had an excellent regulatory effect on the reduction of serum T levels caused by depression (Chen et al., 2015). However, the impact of Chaigui granule on estrogen and its anti-depressant effect has not been studied.

In this study, the anti-depressant effect of Chaigui granule was evaluated by the therapeutic administration to CUMS depression rat model (i.e., modeling followed by drug administration while continuing modeling). Currently, the clinical treatment of depression patients is mostly post-diagnosis pharmacotherapy. Therefore, we used the therapeutic administration method to evaluate the therapeutic administration efficacy of the Chaigui granule to fit the clinical treatment mode better. It is worth noting that the dosage of Chaigui granule in this experiment was obtained based on previous research (Teng et al., 2020). Although the recognized modeling procedure for CUMS lasts for 4 weeks, the current modeling cycle used to establish the CUMS depression model varies (Fu et al., 2018; Li et al., 2019). In this study, behavioral tests were performed on rats on the basis of 3 weeks of modeling, and it was found that the CUMS depression rat model had been successfully replicated after analysis of behavioral data, and then the depressed rats were treated on this basis. By analyzing the final behavioral results, we can find that all the administration groups of Chaigui granule improved the depression-like behavior of CUMS depressed rats.

### 4.1 Effect of chaigui granule on alteration of E2 in the hippocampus of CUMS rats

It is well-known that the hippocampus plays a vital role in depression. Depressed patients are accompanied by varying degrees of hippocampus damage. The effects of E2 in the hippocampus may alter the processing of emotional information and subsequent memory. Also the presence of E2 seems to provide conditions for neurons to create new synapses by increasing dendritic spines; estradiol treatment protects hippocampus synapses from the deleterious effects of acute cortisol elevation (Albert and Newhouse, 2019). The source of E2 in the hippocampus may be the entry of peripheral E2 through blood circulation or the catalytic synthesis of CYP19A1 in the hippocampus (Aizawa et al., 2016). Because sex hormones can enter the brain through the blood-brain barrier by free diffusion (Cui et al., 2013), the hippocampus has an intact steroidase system that generates estradiol (Rebourcet et al., 2020). However, experimental data showed that T levels in the hippocampus were two orders of magnitude lower than T in serum. In contrast, E2 levels in the hippocampus were twice as high as in serum. These results suggest that E2 in the hippocampus be converted mainly by T catalyzed by the CYP19A1 enzyme, which may also be one reason for the lower T levels in the hippocampus than in the serum. The results showed that the Chaigui granule could alleviate the decrease of CYP19A1 and E2 levels in the hippocampus of rats induced by CUMS, demonstrating the regulatory effect of the Chaigui granule on sex hormone levels in the hippocampus. At the same time, the results showed that the positive drugs venlafaxine had no significant impact on the levels of sex hormones in the serum and hippocampus of CUMS depressed rats. However, some studies have shown that the levels of testosterone in the testes of venlafaxine-treated rats were significantly elevated (Fds et al., 2021). Many clinical studies showed that venlafaxine treatment led to the decline of patients’ sexual function. It is considered that low testosterone level is related to venlafaxine treatment (Clayton et al., 2009). Shuganjieyu also had no significant effect on the levels of sex hormones in the serum and hippocampus of CUMS depressed rats. Through literature research, it was found that shuganjieyu combined with paroxetine hydrochloride can increase the level of serum E2 in perimenopausal women (Xi et al., 2021). Still, no study has demonstrated a significant callback effect of shuganjieyu on sex hormone levels in depressed patients.

### 4.2 Effect of chaigui granule on gene and protein expression of related hormones in CYP19A1-E2-ERK signaling pathway in the hippocampus of CUMS rats

First, the mRNA and protein expression results of CYP19A1 can further prove that the Chaigui granule can promote the conversion of T to E2 by regulating CYP19A1 in the hippocampus. Second, Studies have found that E2 can specifically bind to different ERs, activating different cascade signaling pathways and exerting anti-depressant effects. Among them, the MAPK/ERKs signaling pathway is the main pathway for learning, memory, and emotional response in the brain (Yang et al., 2014). Chaigui granule had a regulatory effect on ERα mRNA but did not significantly improve ERα protein levels. mRNA is a template for protein synthesis, whereas transcription and translation are required for protein synthesis. However, the synthesis from gene to protein is a very complex process, and many steps are needed for the final synthesis of protein. Although there is a close relationship between gene expression and protein expression, an increase in gene expression level does not necessarily lead to an increase in protein level. But, the Chaigui granule had a significant effect on ERβ mRNA status and protein expression, which indicated that E2 in the hippocampus might specifically bind ERβ to exert its biological influence demonstrating that the Chaigui granule could improve the regulation of downstream pathways by E2. It also has been shown that injection of ERKs antagonists into the dorsal hippocampus of experimental rats revealed that rats showed significant depression-like behavior (Duman et al., 2007). By analyzing the mRNA levels of relevant molecules on the ERKs pathway, we found that the expression of ERK1/2 and p-ERK1/2 was differentially regulated by the Chaigui granule after the pharmacological intervention, which further demonstrated that the Chaigui granule could promote the specific binding of E2 to its receptor ERβ, which in turn activates the downstream ERK1/2 signaling pathway to exert antidepressant effects.

However, T is also strongly associated with depression, and many studies support that testosterone can improve depressive mood (Osadshiy and Soldatkin, 2022). It has also been shown that depression-like behavior can be induced by decreasing serum and brain testosterone levels in rats (Li et al., 2022). Our previous results also showed a regulatory effect on T levels, for which we also measured the expression of AR mRNA. The results showed that the Chaigui granule could reverse the decrease of AR mRNA expression induced by depression. However, there was no modulation of AR protein in the administration groups. It suggests that Chaigui granule may exert antidepressant efficacy in multiple ways. Besides, Ven had a more substantial modulatory effect on ERβ than ERα, suggesting that venlafaxine could exert an anti-depressant influence by increasing the mRNA contents of ERβ, ERK1, and ERK2. Venlafaxine is an atypical bicyclic anti-depressant that effectively antagonizes the reuptake of 5-HT and NA. However, it was found that venlafaxine strongly regulated ER. After extensive literature research, the anatomical distribution of ER is consistent with the innervation area of 5-HT neurons, and estrogen has a particular regulatory effect on 5-HT. Therefore, venlafaxine may affect ER by regulating 5-HT (Bethea et al., 2002).

This study clarified its anti-depressant mechanism by regulating sex hormone levels and thus ERKs signaling pathway, but there are still shortcomings. In the selection of experimental animals, it was initially thought that the sex hormone levels of male rats were relatively stable; therefore, male rats were used in this study. However, a follow-up study of female rats should be conducted comprehensively and in-depth to fully consider the effect of estrogen changes in female rats during different physiological cycles.

## 5 Conclusion

In this study, it was found that the therapeutic administration of Chaigui granule had significant antidepressant efficacy. Through further studies, it was found that Chaigui granule increased the CUMS-induced decrease in T and E2 contents in serum and hippocampus. After measuring the content of CYP19A1 in the hippocampus, the Chaigui granule was found to promote the conversion of T to E2 in the hippocampus. This may be an important reason why Chaigui granule exerts its antidepressant effect. To test our conjecture, estradiol receptors as well as downstream ERK signaling pathways, were investigated. From the results of RT-qPCR and WB, we concluded that the Chaigui granule could exert an anti-depression effect by promoting the conversion of T to E2 in the hippocampus and activating the ERK signaling pathway downstream of E2.

## Abbreviation

CUMS, A chronic unpredictable mild stress; ELISA, Enzyme-linked immunosorbent assay; RT-qPCR, reverse transcription-quantitative PCR; WB, Western blot; ERK, extracellular regulated protein kinases; T, testosterone; E2, estradiol; CREB, cAMP-response element binding protein; SPF, Sucrose preference test; OFT, Open field test; FST, Forced swimming test; AR, androgen receptors

## Data Availability

The original contributions presented in the study are included in the article/Supplementary Materials, further inquiries can be directed to the corresponding authors.

## Abbreviations

CUMS, A chronic unpredictable mild stress; ELISA, Enzyme-linked immunosorbent assay; RT-qPCR, reverse transcription-quantitative PCR; WB, Western blot; ERK, extracellular regulated protein kinases; T, testosterone; E2, estradiol; CREB, cAMP-response element binding protein; SPF, Sucrose preference test; OFT, Open field test; FST, Forced swimming test; AR, androgen receptors.
