# Supplementary material for: Chaigui granule exerts anti-depressant effects by regulating the synthesis of Estradiol and the downstream of CYP19A1-E2-ERKs signaling pathway in CUMS-induced depressed rats
Source: Front Pharmacol. 2022 Oct 24;13:1005438. doi: 10.3389/fphar.2022.1005438 (PMC9637986; doi:10.3389/fphar.2022.1005438)
Supplement: Supplementary file 1 [file DataSheet1.doc]

Supplementary materials

Xiaoyao San, one of the best-known traditional Chinese medicine prescriptions, was originated from the book of Taiping Huimin Heji Jufang in Song Dynasty (960–1127 AD). And it has been widely used for the treatment of mental disorders in China, such as depression. Based on the clear antidepressant efficacy of Xiaoyao San in the early stage, the research team further conducted systematic research on the prescription through chemical composition analysis, clinical trial observation and pharmacodynamic verification, and then passed the preparation process Optimized and developed a new antidepressant drug Chaigui granule. Through the previous research, we have defined the preparation process of Chaigui granule, established the analysis method of complex chemical components of Chaigui granule by UHPLC-MS technology and identified 95 chemical components in Chaigui granule. At the same time, we also studied the quality standard of Chaigui granule. Firstly, a TLC method was established to identify the *Bupleurum chinense* DC*.* (Apiaceae), *Angelica sinensis* (Oliv.) Diels (Apiaceae), [*Paeonia lactiflora*](https://www.sciencedirect.com/topics/pharmacology-toxicology-and-pharmaceutical-science/paeonia-lactiflora)Pall. (Paeoniaceae) and *Glycyrrhiza uralensis Fisch. ex DC.* (Fabaceae) in Chaigui granule. Secondly, the fingerprint of Chaigui granule was established, and 12 chemical components in Chaigui granule were identified. It provides a basis for the material basis of Chaigui granule. A HPLC method for the simultaneous determination of four saikosaponins in Chaigui granule was established with the total amount of saikosaponins a, b1, b2 and d as the quality control index. A HPLC method for the determination of paeoniflorin was established with paeoniflorin as a quality control index. The quality of Chaigui granule was controlled by measuring the content of the above components. Finally, the durability and stability of Chaigui granule were also investigated and studied. The chaigui granule which we used in this research were achieved the quality demands.


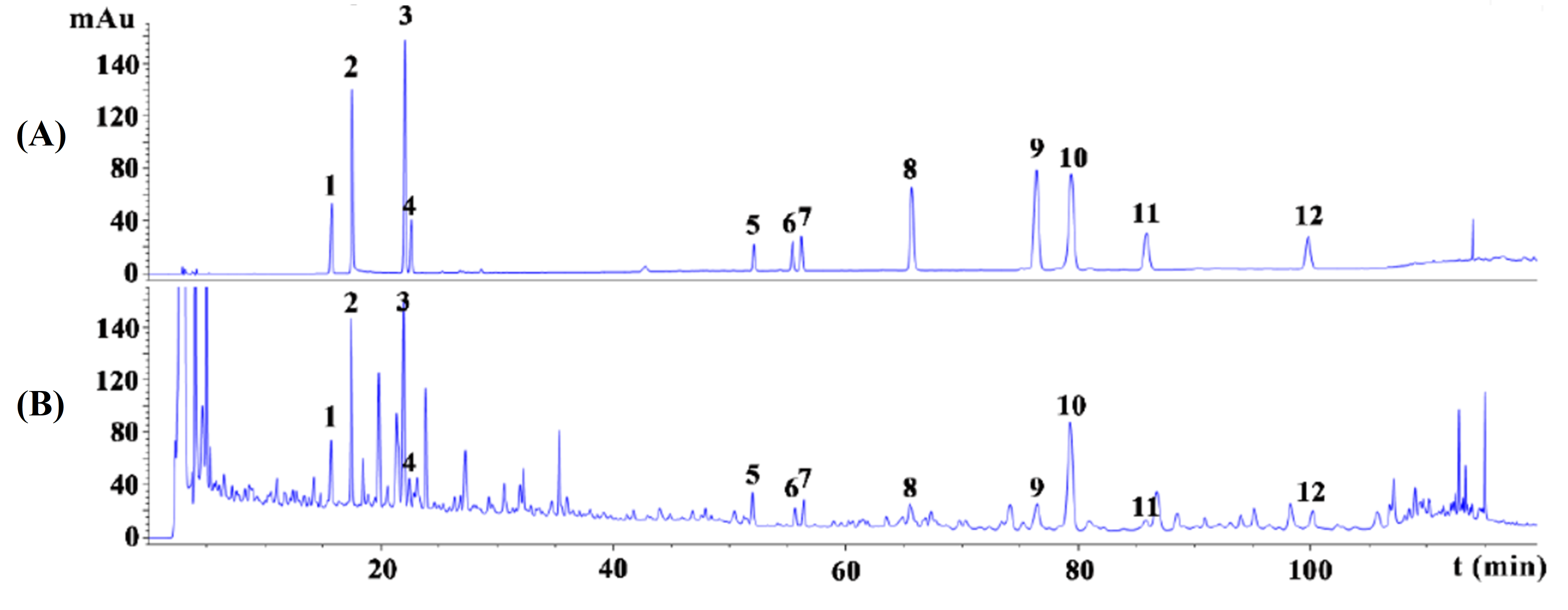


**Supplementary Figure 1.** Chromatogram of reference substance (A) and Chaigui granule (B)

1. Albiflorin；2-Paeoniflorin；3-Liquiritin；4-Ferulic acid；5-Monoammonium Glycyrrhizinate；6-Saikosaponin A；7-Saikosaponin B2；8-Atractylenolide III；9-Imperatorin；10-Ligustilide；11-Atractylenolide I；12-2-Atractylenolide)

**Supplementary Table 1.** Content of Saikosaponin (a, b_2_, b_1_, d) in Three Batches of Chaigui granule

| Batch | SS a (%) | SS b_2_ (%) | SS b_1_ (%) | SS d (%) | Total content (%) |
| --- | --- | --- | --- | --- | --- |
| 1 | 0.24 | 0.40 | 0.05 | 0.04 | 0.73 |
| 2 | 0.27 | 0.38 | 0.04 | 0.08 | 0.77 |
| 3 | 0.23 | 0.40 | 0.06 | 0.05 | 0.71 |

**Supplementary Table 2.** Paeoniflorin Content in Three Batches of Chaigui granule

| Batch | Paeoniflorin content (%) |
| --- | --- |
| 1 | 0.654 |
| 2 | 0.662 |
| 3 | 0.608 |
